# The role of the *Candida* biofilm matrix in drug and immune protection

**DOI:** 10.1016/j.tcsw.2023.100111

**Published:** 2023-10-09

**Authors:** Sumita Roy, Neil A.R. Gow

**Affiliations:** MRC Centre for Medical Mycology, University of Exeter, Geoffrey Pope Building, Stocker Road, Exeter, EX4 4QD, UK

**Keywords:** AFR, Biofilm, β-1, 3 glucan, Mannan, Persister cell

## Introduction

**Fig. 1:**
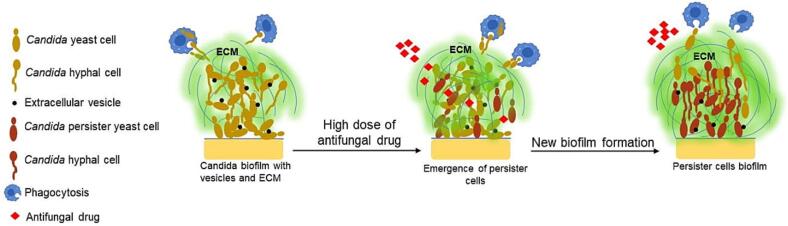
Schematic diagram showing Candida biofilm with extracellular vesicles (EV) (Black dot) and extracellular matrix (ECM). ECM protects Candida from both antifungal drugs by reducing access to the fungal cells and also protects against phagocytosis (Blue) by macrophages and other phagocytes. In presence of high dose of antifungal drugs (Red), persister *Candida* cells (Brown) emerge that can reseed the biofilm with viable cells post antifungal therapy.

*Candida albicans, Candida auris* and *Candida tropicalis* are three fungal pathogens that WHO recently reported as requiring urgent attention and additional resources for research and development (World Health Organisation, 2022). *Candida* species often form biofilms on the surfaces of tissues or medical devices, such as catheters and heart values and these represent reservoirs of infection that are difficult to eradicate using conventional antifungal treatment (Junqueira and Mylonakis, 2023, Nett and Andes, 2020, Ramage et al., 2023). The biofilms compromise the protective capacity of sentinel activities of the host immune system, and they render the fungal biomass resistant to most clinically relevant antifungal drugs. *Candida albicans* biofilms contain yeast, hyphae and a complex extracellular matrix (ECM) whilst other species form biofilms with a simpler array of cell types. The biofilm is a three dimensional structure composed of a foundation basal layer of yeast cells that is tightly adhered to a biological or non-biological surface from which a proliferation of branching and budding hyphae and yeast cells are seeded and which is encased in covering of the ECM (Atiencia-Carrera et al., 2022, Gulati and Nobile, 2016). The presence of the ECM radically alters the physiology of the fungal cells and confers protective properties that severely compromise the ability of immune cells and administration of antifungal drugs to kill the fungus (Ajetunmobi et al., 2023) (Fig. 1). We review here the properties of this ECM and how this influences the drug resistant phenotype of *Candida* cells in biofilms and help protect cells against immune phagocytes.

The drug resistance phenotype of the *Candida* biofilms is related to a number of properties of the ECM – its impermeability, ability to sequester and immobilise many drugs, and the presence of drug resistance persister cells within the biofilm. Persister cells represent a minority of cells of the biofilm, but the proportion may increase after exposure to high doses of antifungal drugs (Fig. 1). These are a non-growing, metabolically quiescent subpopulation of the biofilm cells that are able to survive high doses of antimicrobial drugs (Li et al., 2015, Wuyts et al., 2018). These dormant cells exhibit properties that are similar to glucose-starved planktonic cells whose physiology enables them to survive challenge with fungicidal drugs by inducing stress tolerance pathways and protective levels of internal glycogen and trehalose (Wuyts et al., 2018). Persister cells of the biofilm may increase the production of ECM materials and induce enzyme activities that promote their survival. For example, the Bgl2 glucanosyltransferase, the exoglucanase Xog1, and the signalling proteins KRE1 and SKN1 were shown to be upregulated in persister cells. These proteins, along with Extracellular Vesicles (EVs), are involved in ECM production (Li et al., 2015).

## Biofilm composition and function in drug sensitivity

*Candida* biofilm matrix is composed approximately of 25 % carbohydrate, 55 % protein, 15 % lipid and 5 % nucleic acid (Mitchell et al., 2016, Pierce et al., 2017). The carbohydrate component of the biofilm contains similar polysaccharides as found in cell wall; however, the macromolecular structures of the cell wall and biofilm polysaccharides are distinct in their fine structure and they may be actively modified after they are secreted from the cell into the ECM (Mitchell et al., 2016). A major contributor of biofilm ECM, including 45 % of the ECM protein is derived from Extracellular Vesicles (EVs), and biofilm EVs are distinct in composition from those generated by planktonic cells (Zarnowski et al., 2018). Mutants in the ESCRT pathway required for vesicle biogenesis and secretion prevented EVs entering into the biofilm, and this prevented ECM synthesis and resulted in increased sensitivity to fluconazole (Zarnowski et al., 2018). It is interesting that AmBisome liposomal vesicles, have been shown to be able to transit the intact fungal cell wall (Walker et al., 2018), and so vesicular carriers seem to be important for the synthesis of the ECM and may provide opportunities in the treatment with antifungal drugs encapsulated in liposomes.

Using NMR, it was shown that mannans and β-glucan two of the major polysaccharides identified in *Candida* EVs (Zarnowski et al., 2018). The biofilm matrix contains α-1,2- mannan, α −1,6 mannan and β-1,6 glucan which are greatly enriched in abundance compared to the underlying cell wall. Mannan is less rigid than chitin or β1,3 glucan (Gow & Lenardon, 2022), but makes the matrix complex antifungal drug resistant by decreasing permeability of the biofilm (Walker et al., 2018). In addition, the β-1,3 glucan, β −1,6 glucan, and α-1,2-branched α −1,6 mannan components of the ECM can sequester drugs such as amphotericin B, anidulafungin, and flucytosine via non-covalent binding, thereby reducing their efficacy (Fig. 1) (Mitchell et al., 2016, Nett and Andes, 2020). In addition, mutants in *endo*-β-D-glucosidase (Sun41), that degrades ECM increases matrix, affects the sensitivity of the fungus to antifungal drugs such as caspofungin (Norice et al., 2007). A range of other genes that are key to the synthesis of β-1,3 glucan all have a common phenotype of enhanced susceptibility to fluconazole (Mitchell et al., 2013, Taff et al., 2012).

In *Candida auris* amplification of *ALS4* copy number enhances biofilm and adherence (Bing et al., 2023) – two aspects of the fungus which is blocked by treatments that inhibit amyloid protein function (Malavia-Jones et al., 2023). Thus both cell wall, ECM polysaccharides and proteins all contribute to the structure of the ECM and to its antifungal drug retarding properties.

To date, it is not clear if there are any changes to the cell wall component in the persister cells although it is highly likely since growth rate affects cell wall composition (Gow & Lenardon, 2022). Supporting this, it is known that components of the cell-wall integrity pathways (XOG1, BGl1, SUN41, SCW11 and PSA2) are up-regulated in persister cells in the presence of Amphotericin B induced oxidative stress (Li et al., 2015).

A genome wide screen identified six “master transcriptional regulators” -Efg1, Tec1, Bcr1, Ndt80, Brg1 and Rob1, for biofilm formation (Cavalheiro and Teixeira, 2018, Nobile et al., 2012). Each regulator plays a visible roles in regulating biofilm structure. For example, only the master regulator Bcr1 and downstream cell wall proteins (Als1, Als3 and Hwp1) are required for the first crucial attachment step of biofilm formation to surfaces. However, Bcr1 is not required for hyphal formation but it is required for hyphal attachment in biofilms associated with oropharyngeal candidiasis (Fanning et al., 2012).

## Advances in the treatment of fungal biofilms

A range of advances have focussed on treating fungal biofilms to improve drug efficacy (Junqueira & Mylonakis, 2023). A promising antifungal anti-biofilm drug, turbinmicin, has been shown to disrupt the extracellular vesicles production and eliminate the extracellular matrix of *Candida* biofilm. Turbinmicin is likely to target the Sec14p-a phosphatidylinositolphosphatidyl-choline transfer protein involved in the vesicle trafficking, and thereby biofilm formation (Zhao et al., 2021).

Administration of membrane active antimicrobial peptides such as gH625 (Galdiero et al., 2020), and a scorpion venom ToAP2 peptide (do Nascimento Dias et al., 2020) inhibit ECM formation in biofilm, reduced the number of persister cells and increased antifungal drug susceptibility to a number of antifungals (Galdiero et al., 2020).These peptides increased the permeability of cell membrane and penetration through the ECM. Surfaces can also be created or treated to impede biofilm establishment, for example using surface functionalization with antifungals and the use of nanoparticles that incorporate inhibitory polymers or antifungals. These methods have shown promising results in disruption and dispersing biofilms (Vera-Gonzalez & Shukla, 2020). Combinational therapies in which agents that improve penetration and permeability of membranes may also have useful applications in the treatment of *Candida* biofilms. Similarly, do Nascimento Dias et al (2020) reported that when ToAP2 peptide was used in combination with fluconazole and Amphotericin B, there was increase in efficacy of both molecules (do Nascimento Dias et al., 2020). Furthermore, the membranotropic peptide-gH625 used in combination with fluconazole and 5-fluorocytosine was able to efficiently eradicate biofilms and persistor cells (Galdiero et al., 2020). Analogues of diazaspiro-decanes have also been shown to be bioactive biofilm inhibitors (Pierce et al., 2015). Even low micromolar concentrations of compounds with a common biaryl amide structure inhibited *C. albicans* biofilm formation and filamentous growth.

## Conclusions

In summary, *Candida* biofilms remain a significant clinical problem because of their ability to restrict access to both antifungal drugs and immune cells. It is clear that the ECM that encases growing and non-growing persister cells is distinct in composition to the cell wall and has important clinically relevant properties. These studies also demonstrate that the cell wall of fungi is not the only outer structure that effects drug permeability and sensitivity (Casadevall & Gow, 2022). The ECM can bind and sequester drugs and represent a permeability barrier. It also provides a protective microenvironment in which metabolically quiescent persister cells can survive periods of antifungal drugs administration, only to emerge and proliferate when drug levels in the bloodstream subside.

Future studies must focus on understanding the role of each component of the biofilm structure and how they influence antifungal drug resistance. This knowledge will be pivotal in treating fungal infections and the roles of key components of fungal walls and matrices.

## CRediT authorship contribution statement

**Sumita Roy:** Writing – original draft, Investigation, Conceptualization. **Neil A.R. Gow:** Writing – original draft, Supervision, Funding acquisition, Conceptualization.

## Declaration of Competing Interest

The authors declare that they have no known competing financial interests or personal relationships that could have appeared to influence the work reported in this paper. Given his role as Editor-in-Chief, Neil Gow had no involvement in the peer review of this article and has no access to information regarding its peer review. Full responsibility for the editorial process for this article was delegated to Wenxia Fang.
